# Correction: Mountain gorillas maintain strong affiliative biases for maternal siblings despite high male reproductive skew and extensive exposure to paternal kin

**DOI:** 10.7554/eLife.89419

**Published:** 2023-05-18

**Authors:** Nicholas M Grebe, Jean Paul Hirwa, Tara S Stoinski, Linda Vigilant, Stacy Rosenbaum

**Keywords:** Other

Grebe NM, Hirwa JP, Stoinski TS, Vigilant L, Rosenbaum S. 2022. Mountain gorillas maintain strong affiliative biases for maternal siblings despite high male reproductive skew and extensive exposure to paternal kin. *eLife* **11**:e80820. doi: 10.7554/eLife.80820.

Published 22 September 2022

We were contacted by a colleague following the publication of our paper, who made us aware of a potential bias introduced by our treatment of random intercepts in our statistical models. In our models of dyadic behavior, the assignment of an individual to the “ID1” versus “ID2” position in each row of data was determined alphabetically, as our target behaviors were not directional. ID1 and ID2 were included as random intercepts, in addition to dyad ID, in all models. Consequently, certain individuals with names towards the beginning of the alphabet were disproportionately likely to occupy the ID1 position, and conversely, those with names towards the end of the alphabet were disproportionately likely to occupy the ID2 position. This bias has potential consequences for the estimation of random effect variances for each term, and thus our reported results in both the main text and the appendices.

To address this possible bias, we randomly shuffled the assignment of ID1 and ID2 in each row across 100 replicates of our dataset, ran our focal models with specifications identical to the original paper on each of these replicates, and reported results from a pooled summary in the corrected results section. This approach was inspired by the modelling strategy of van Leeuwen et al. (2012):

van Leeuwen, E.J C., Cronin, K.A., Haun, D.B.M., Mundry, R., & Bodamer, M.D. (2012). Neighbouring chimpanzee communities show different preferences in social grooming behaviour. *Proceedings of the Royal Society B: Biological Sciences*, 279(1746), 4362–4367.

Because this new approach affects the calculated random effect variances in every model, all of the coefficients and *p-*values we report in our results section, and in our appendices, have changed slightly. However, as the differences are slight, only one minor qualitative claim has changed in the new results section (concerning differences between full and maternal siblings in play rates). None of the substantive conclusions in our discussion have changed.

Regarding figures, all box and dot plot figures remain unchanged, as the underlying data remains unchanged. This includes Figure 1 and both its supplements; Figure 4A; Figure 5A and its supplement; Appendix 4—figure 1; and Appendix 4—figure 2.

Figures that depict model-estimated slopes have all slightly changed with the new models. This includes Figure 2; Figure 3; Figure 4B; Figure 5B; and Appendix 3—figure 1. As with the modified results, the qualitative conclusions remain the same.

We are grateful to Marina Cords for alerting us to this issue.

**Corrected text in Results:**

**Results**

*Affiliative behaviors*

In our full sample of 1934 unique dyads spanning 7832 dyad-years, full siblings (n=43 dyads) and maternal siblings (n=101 dyads) played and groomed each other significantly more than did paternal siblings (n=555 dyads) or non-siblings (n=1235 dyads; all comparisons p<0.05; Figure 1A and B). Age differences (in our sample, mean: 5.85 years; SD: 4.53 years; range: 0–23.5 years) interacted with relatedness in predicting grooming (p=0.017), but not play (p=0.427). Play consistently dropped for siblings and non-siblings alike as age differences increased (γ ranging from –0.31 to –0.37, all p<0.001; Figure 2A). By contrast, grooming rates were relatively unrelated to age differences between partners (γ ranging from –0.09 to 0.00, all p>0.05; Figure 2B).

The corrected Figure 2 is shown below:

**Figure 2. Estimated rates of play (A)and grooming (B)across a range of age differences, separated by relatedness category.** Bars represent 95% confidence intervals for rates of behavior at a given age difference.

**Figure 1:**
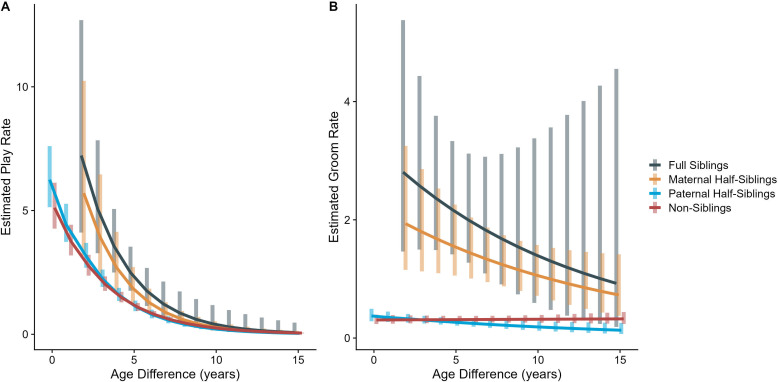


The originally published Figure 2 is shown below for reference:

**Figure 2. Estimated rates of play (A)and grooming (B)across a range of age differences, separated by relatedness category.** Bars represent 95% confidence intervals for rates of behavior at a given age difference.

**Figure 2:**
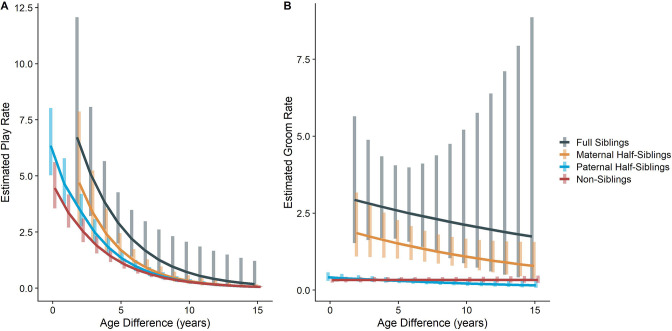


Male-male dyads (n=503) played more than either mixed-sex (n=977) or female-female dyads (n=454); conversely, female-female dyads groomed each other more than either mixed-sex or male-male dyads (all p<0.001; Figure 1C and D). These patterns for play and grooming were strongly moderated by age differences (ps<0.006) and were significantly, albeit more weakly, moderated by relatedness (p=0.012 and 0.045, respectively). Play dropped rapidly with increasing age differences (*γ* = –0.37 to –0.30) for all sex configurations (all p<0.001; Figure 3A). Grooming was steadily low in male-male and mixed-sex dyads (*γ* = –0.02 and –0.03, p>0.32), though it dropped with increasing age differences in female-female dyads (*γ* = –0.11, p<0.001), such that differences between sex categories became indistinguishable after approximately a 10-year age difference (Figure 3B). Play was consistently highest among male-male dyads within all relatedness categories, with the exception of maternal siblings, who exhibited more comparable rates of play among male-male and mixed-sex dyads (Figure 1—figure supplement 1). Grooming was also consistently highest among female-female dyads of all types, though the magnitude of this difference varied between relatedness categories (Figure 1—figure supplement 2).

The corrected Figure 3 is shown below:

**Figure 3. Estimated rates of play (A)and grooming (B)across a range of age differences, separated by sex category.** Bars represent 95% confidence intervals for rates of behavior at a given age difference.

**Figure 3:**
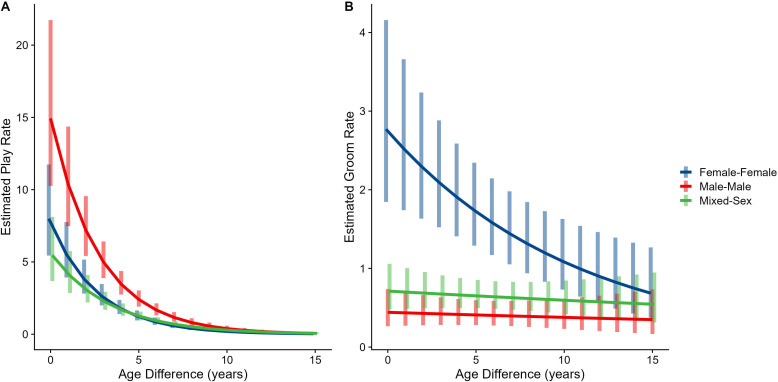


The originally published Figure 3 is shown below for reference:

**Figure 3. Estimated rates of play (A)and grooming (B)across a range of age differences, separated by sex category.** Bars represent 95% confidence intervals for rates of behavior at a given age difference.

**Figure 4:**
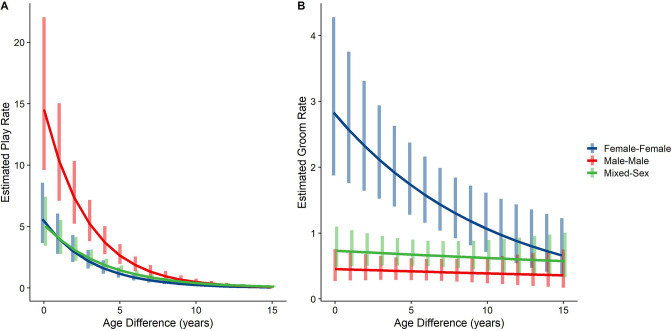


When restricting analyses to dyads who lived in the same social group during the first year of the younger partner’s life (n=6724 dyad-years), results for affiliative behavior remained qualitatively similar: full and maternal siblings played and groomed more than paternal siblings or non-siblings (see Appendix 1—tables 1 and 2). Interactions between relatedness categories and sex makeup weakened and were no longer significant (for play: p=0.387; for grooming: p=0.347); sex category × age difference interactions remained significant for grooming (p<0.001) and for play (p=0.031). Additionally, all reported results were robust to the inclusion of average age of the dyad as a covariate (Appendix 3).

*Time spent in proximity*

The time dyads spent in close proximity (<2 m) with each other also varied between relatedness categories (p<0.001), with maternal siblings and full siblings once again spending more time near each other than non-siblings, who themselves spent more time in close proximity than paternal siblings did (all comparisons p<0.001; Figure 4A). However, these patterns too were moderated by age differences (p<0.001). Proximity decreased with increasing age differences in maternal siblings and paternal siblings (*γ* = –0.08 and –0.09, p<0.001), but did not decrease significantly in full siblings or non-siblings (*γ* = –0.04 and 0.01, p>0.29). Thus, while all classes of siblings spent more time near each other than non-siblings when near in age, even when adjusting for their mother’s presence, this distinction was partially reversed at large age differences, when paternal siblings spent much less time near each other than any other dyad category (Figure 4B).

The corrected Figure 4 is shown below:

**Figure 4. Box and dot plots (A)and estimated trends across a range of age differences (B)for the time gorilla dyads spent in close proximity, separated by relatedness category.** Bars in (B) represent 95% confidence intervals for rates of proximity at a given age difference.

**Figure 5:**
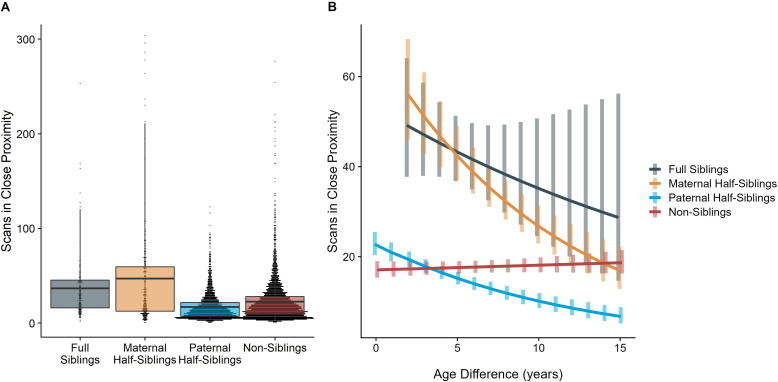


The originally published Figure 4 is shown below for reference:

**Figure 4. Box and dot plots (A)and estimated trends across a range of age differences (B)for the time gorilla dyads spent in close proximity, separated by relatedness category.** Bars in (B) represent 95% confidence intervals for rates of proximity at a given age difference.

**Figure 6:**
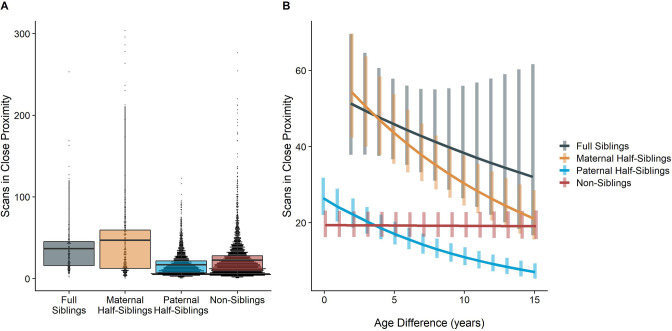


When restricting analyses to dyads who lived in the same social group during the first year of the younger partner’s life, results for proximity remained consistent: full and maternal siblings spent more time together than paternal siblings or non-siblings, though this difference was reversed at large age differences in the same manner as our primary model (see Appendix 1—tables 1 and 2 for full results).

*Competitive behaviors*

Neither relatedness nor sex category on their own significantly predicted rates of aggressive behavior (p=0.876 and 0.838, respectively). However, our model did reveal a significant sex makeup ×relatedness interaction term (p=0.025; Figure 5A). Decomposing this interaction, among female-female and male-male dyads, there were no statistically significant contrasts between relatedness categories. In mixed-sex dyads, non-siblings engaged in substantially more aggression than any sibling category (all p<0.050). While sex category also interacted with age differences in predicting aggression (p=0.009), marginal trends were consistently negative (*γ* = –0.14 to –0.08, p<0.001), such that, across sex categories, dyads more distant in age engaged in less aggression than dyads closer in age (Figure 5B). All reported results were robust to the inclusion of average age of the dyad as a covariate (Appendix 3).

The corrected Figure 5 is shown below:

**Figure 5. Box and dot plots (A)and estimated trends across a range of age differences (B)for aggression within gorilla dyads, separated by relatedness and sex category.** Bars in (B) represent 95% confidence intervals for rates of aggression at a given age difference.

**Figure 7:**
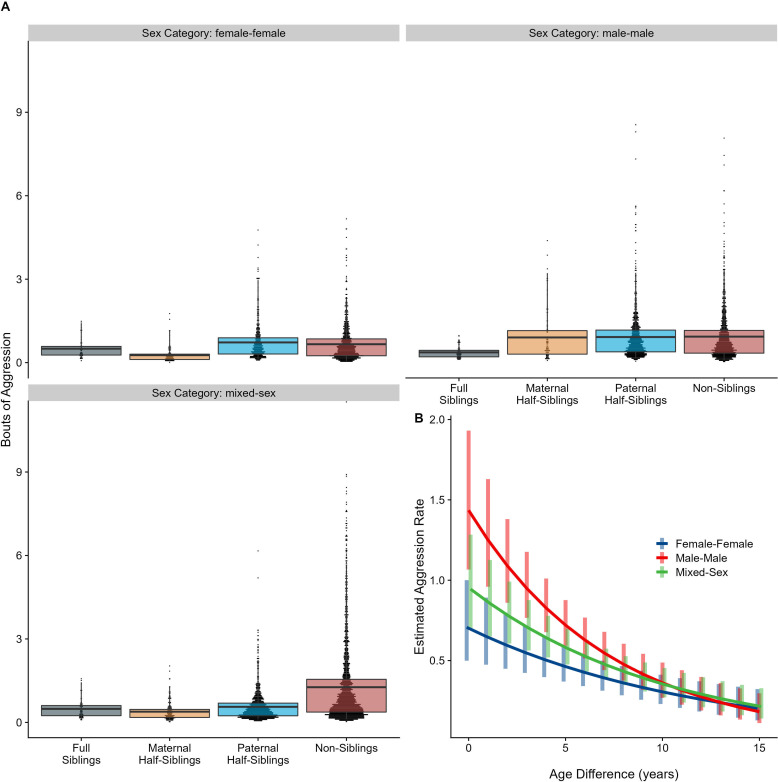


The originally published Figure 5 is shown below for reference:

**Figure 5. Box and dot plots (A)and estimated trends across a range of age differences (B)for aggression within gorilla dyads, separated by relatedness and sex category.** Bars in (B) represent 95% confidence intervals for rates of aggression at a given age difference.

**Figure 8:**
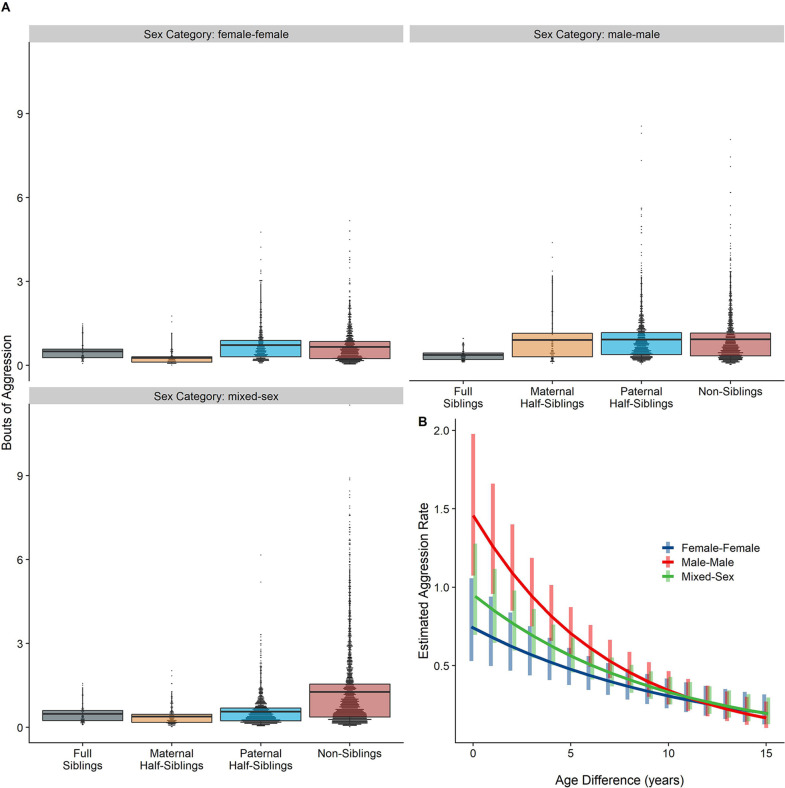


Unlike results for affiliative behavior, when restricting analyses to dyads who lived in the same social group during the first year of the younger partner’s life, results for aggressive behavior qualitatively shifted. Relatedness and sex categories no longer significantly interacted (p=0.444), and the significant pairwise differences between siblings and non-siblings in mixed-sex dyads shrank markedly and were no longer significant (all p>0.60). Age difference remained a highly significant predictor of aggression (p<0.001). Inspection of our data confirmed that much aggression specifically occurred in the context of females transferring into groups–that is, when females first encountered unrelated, unfamiliar males–accounting for non-significant pairwise differences among male-female dyads with early-life familiarity (Figure 5—figure supplement 1). See Appendix 1—tables 1 and 2 for full results.

**Original text in Results:**

**Results**

*Affiliative behaviors*

In our full sample of 1934 unique dyads spanning 7832 dyad-years, full siblings (n=43 dyads) played and groomed each other significantly more than did paternal siblings (n=555 dyads) or non-siblings (n=1235 dyads; all comparisons p<0.001; Figure 1A and B). Maternal siblings (n=101 dyads) played significantly less than full siblings but they groomed at comparable rates. Age differences (in our sample, mean: 5.85 years; SD: 4.53 years; range: 0–23.5 years) interacted with relatedness in predicting grooming (p=0.035), but not play (p=0.577). Play consistently dropped for siblings and non-siblings alike as age differences increased (γ ranging from –0.28 to –0.23, all p<0.001; Figure 2A). By contrast, grooming rates were relatively unrelated to age differences between partners (γ ranging from –0.07 to –0.01, all p>0.05; Figure 2B).

Male-male dyads (n=503) played more than either mixed-sex (n=977) or female-female dyads (n=454); conversely, female-female dyads groom each other more than either mixed-sex or male-male dyads (all p<0.001; Figure 1C and D). These patterns for play and grooming were strongly moderated by age differences (ps <0.002) and were significantly, albeit more weakly, moderated by relatedness (p=0.025 and 0.036, respectively). Play dropped rapidly with increasing age differences (*γ*=–0.34––0.26) for all sex configurations (all p<0.001; Figure 3A). Grooming was steadily low in male-male and mixed-sex dyads (*γ*=–0.02––0.02, p>0.50), though it dropped with increasing age differences in female-female dyads (*γ*=–0.09, p=0.002), such that differences between sex categories became indistinguishable after approximately a 10 year age difference (Figure 3B). Play was consistently highest among male-male dyads within all relatedness categories, with the exception of maternal siblings, who exhibited more comparable rates of play among male-male and mixed-sex dyads (Figure 1—figure supplement 1). Grooming was also consistently highest among female-female dyads of all types, though the magnitude of this difference varied between relatedness categories (Figure 1—figure supplement 2).

When restricting analyses to dyads who lived in the same social group during the first year of the younger partner’s life (n=6724 dyad-years), results for affiliative behavior remained qualitatively similar: full and maternal siblings played and groomed more than paternal siblings or non-siblings (see Figure 1—figure supplements 1 and 2, Appendix 1—tables 1 and 2). Interactions between relatedness categories and sex makeup weakened and were no longer significant (for play: *P*=0.555; for grooming: p=0.243); sex category×age difference interactions remained highly significant for grooming (p<0.001) and were marginally significant for play (p=0.070). Additionally, all reported results were robust to the inclusion of average age of the dyad as a covariate (Appendix 3—Tables 1 and 2).

*Time spent in proximity*

The time dyads spent in close proximity (<2 m) with each other also varied between relatedness categories (p<0.001), with maternal siblings and full siblings once again spending more time near each other than non-siblings, who themselves spent more time in close proximity than paternal siblings did (all comparisons p<0.001; Figure 4A). However, these patterns too were moderated by age differences (p<0.001). Proximity decreased with increasing age differences in maternal siblings and paternal siblings (*γ*=–0.08––0.09, p<0.001), but did not decrease significantly in full siblings or non-siblings (*γ*=–0.04–0.01, p>0.26). Thus, while all classes of siblings spent more time near each other than non-siblings when near in age, even when adjusting for their mother’s presence, this distinction was partially reversed at large age differences, when paternal siblings spent much less time near each other than any other dyad category (Figure 4B).

When restricting analyses to dyads who lived in the same social group during the first year of the younger partner’s life, results for proximity remained consistent: full and maternal siblings spent more time together than paternal siblings or non-siblings, though this difference was reversed at large age differences in the same manner as our primary model (see Appendix 1—tables 1 and 2 for full results).

*Competitive behaviors*

Neither relatedness nor sex category on their own significantly predicted rates of aggressive behavior (p=0.217 and 0.625, respectively). However, our model did reveal a significant sex makeup×relatedness interaction term (p=0.017; Figure 5A). Decomposing this interaction, among female-female and male-male dyads, there were no statistically significant contrasts between relatedness categories. In mixed-sex dyads, non-siblings engaged in substantially more aggression than any sibling category (all p<0.050). While sex category also interacted with age differences in predicting aggression (p=0.005), marginal trends were consistently negative (*γ*=–0.14––0.09, p<0.001), such that, across sex categories, dyads more distant in age engaged in less aggression than dyads closer in age (Figure 5B). All reported results were robust to the inclusion of average age of the dyad as a covariate (Appendix 3—Tables 1 and 2).

Unlike results for affiliative behavior, when restricting analyses to dyads who lived in the same social group during the first year of the younger partner’s life, results for aggressive behavior qualitatively shifted. Relatedness and sex categories no longer significantly interacted (p=0.553), and the significant pairwise differences between siblings and non-siblings in mixed-sex dyads shrank markedly and were no longer significant (all p>0.44). Age difference remained a highly significant predictor of aggression (p=0.004). Inspection of our data confirmed that much aggression specifically occurred in the context of females transferring into groups–i.e., when females first encountered unrelated, unfamiliar males–accounting for non-significant pairwise differences among male-female dyads with early-life familiarity (Figure 5—figure supplement 1). See Appendix 1—tables 1 and 2 for full results.

